# Circular RNA circWWC3 augments breast cancer progression through promoting M2 macrophage polarization and tumor immune escape via regulating the expression and secretion of IL-4

**DOI:** 10.1186/s12935-022-02686-9

**Published:** 2022-08-22

**Authors:** Yang Zheng, Shuguang Ren, Yu Zhang, Sihua Liu, Lingjiao Meng, Fei Liu, Lina Gu, Ning Ai, Meixiang Sang

**Affiliations:** 1 Tumor research Institute, The Fourth Hospital of Hebei Medical University, Shijiazhuang, 050017 Hebei People’s Republic of China; 2 Key Laboratory for Tumor diagnosis, Prevention and Therapy in Hebei Province, Shijiazhuang, 050017 Hebei People’s Republic of China; 3 Animal Center, The Fourth Hospital of Hebei Medical University, Shijiazhuang, 050017 Hebei People’s Republic of China; 4 Radiology Department, The Fourth Hospital of Hebei Medical University, Shijiazhuang, 050017 Hebei People’s Republic of China

**Keywords:** Breast cancer, Tumor-associated macrophages, circWWC3, IL-4, PD-L1

## Abstract

Interaction between tumor cells and tumor microenvironment (TME) is critical to promote tumor progression and metastasis. As the most abundant immune cells in TME, macrophages can be polarized into M2-like tumor-associated macrophages (TAMs) which further promote tumor progression. However, to date, the molecular mechanisms of TAM polarization in TME are still largely unknown. In the present study, we revealed that circular RNA circWWC3 could up-regulate the expression and secretion of IL-4 in breast cancer cells. Enhanced secretion of IL-4 from breast cancer cells could augment the M2-like polarization of macrophages in TME, which further promotes the migration of breast cancer cells. In addition, increased secretion of IL-4 from breast cancer cells could induce the expression PD-L1 in M2 macrophages. Moreover, up-regulated IL-4 also enhanced the expression of PD-L1 in breast cancer cells, which further facilitates breast cancer immune evasion. Though analyzing the expression of circWWC3, IL-4, PD-L1, and CD163 in 140 cases of breast cancer tissues, we found that high expression of circWWC3 was associated with poor overall survival and disease-free survival of breast cancer patients. Breast cancer patients with circWWC3^high^/PD-L1^high^ breast cancer cells and CD163^high^ macrophages had a poorer overall survival and disease-free survival. Conclusively, circWWC3 might augment breast cancer progression through promoting M2 macrophage polarization and tumor immune escape via regulating the expression and secretion of IL-4. CircWWC3 might be a potential therapeutic target in breast cancer.

**Supplementary Information:**

The online version contains supplementary material available at 10.1186/s12935-022-02686-9.

## Introduction

Crosstalk between tumor and immune cells is critical to promote tumor progression and metastasis [1]. As the most abundant immune cells in tumor microenvironment (TME), macrophages can respond to various factors produced by tumor cells in the TME [2]. Macrophages can be polarized into two phenotypes, namely M1 and M2, depending on the environmental stimulation of different factors, forming a type of heterogeneous immune cells that exert immune-stimulatory or immune-suppressive effects. Generally, M1 polarized macrophages can express high-level pro-inflammatory cytokines to eliminate tumor cells, whereas M2 polarized macrophages are characterized by secreting a large number of anti-inflammatory cytokines, growth factors, inhibitory molecules such as PD-L1 and PD-L2, to promote tumor cell progression [3–6]. Tumor-associated macrophages(TAMs) are mainly considered to be the M2 polarized macrophages that play an immunosuppressive function and promote the angiogenesis, proliferation, invasion and metastasis of tumor cells, thereby closely associated with the poor prognosis of tumor patients [7]. However, to date, the molecular mechanisms of TAM polarization in TME remain largely unknown.

Circular RNAs (circRNAs) are a novel type of endogenous RNAs that are characterized by a covalently closed loop structure without 5’-end cap and 3’-end poly A tail, thus are highly stable and resistant to RNA exonucleases [8–12]. Accumulating evidences showed that circRNAs regulated tumor development and progression by acting as oncogenes or tumor suppressors via affecting a variety of signaling pathways [13]. In our previous study, we identified a novel circRNA hsa_circ_0001910 (circWWC3) which was directly induced by ZEB1. CircWWC3 was derived from exon2 to exon8 (825 nt) from the WWC3 gene flanked by long introns on either side, and promoted breast cancer progression through targeting Ras signaling pathways [14]. However, the molecular mechanism of circWWC3 in breast cancer progression is still largely undiscovered.

In the present study, we revealed that circWWC3 could up-regulate the expression and secretion of IL-4 in breast cancer cells. Enhanced secretion of IL-4 from breast cancer cells could augment the M2-like polarization of macrophages in TME, which further promoted the migration of breast cancer cells. In addition, increased IL-4 expression and secretion could induce the expression of PD-L1 in breast cancer cells and M2 macrophages, which further facilitated breast cancer immune evasion. In breast cancer tissues, high expression of circWWC3 was associated with poor overall survival and disease-free survival of breast cancer patients. Breast cancer patients with circWWC3^high^/PD-L1^high^ breast cancer cells and CD163^high^ macrophages had a poorer overall survival and disease-free survival. Thus, circWWC3 might augment breast cancer progression through promoting M2 macrophage polarization and tumor immune escape via regulating the expression and secretion of IL-4. The purpose of our study was to explore the molecular mechanism of circWWC3 in M2 macrophage polarization and tumor immune escape in breast cancer.

## Materials and methods

### cDNA microarray

Total RNA was extracted from MDA-MB-231 cells transfected with control siRNA or si-circWWC3. Agilent SurePrint G3 Human Gene Expression v3 8 × 60 K Microarray was used to identify differentially expressed genes. The nucleic acid preparation and microarray hybridization process were carried out based on Agilent’s protocols.

### Tumor specimens

Human breast cancer tissues and adjacent normal tissues were collected from Shanghai Outdo Biotech Company. All patients did not receive preoperative chemotherapy and radiation therapy. The human tissues were obtained with informed consent, and our study was approved by the Research Ethics Committee of Shanghai Outdo Biotech Company.

### Cell culture, RNA extraction, reverse transcription-quantitative real-time PCR (qRT-PCR)

All cell lines were cultured in DMEM (GIBCO, USA) added 10% fetal heat-inactivated bovine serum (GIBCO, USA) and 1% Penicillin-Streptomycin Solution (100×). Cells were grown in a 37 °C, 5% CO_2_ incubator. Total RNA was extracted by TRIzol Reagent (Invitrogen, USA). The cDNA was synthesized from total RNA using GoScript™ Reverse Transcription System (Promega, USA). qRT-PCR was performed by using GoTaq® qPCR Master Mix (Promega) in ABI QuantStudio™ 6 Flex. The primers in Additional file 1: Table S1. The expression levels were calculated according to the comparative C_T_ method (ΔΔC_T_).

### Western blot

Proteins were separated by 10% SDS-PAGE, and then transferred onto PVDF membranes (Millipore, Billerica, MA, USA). Mouse IL-4 monoclonal antibody (proteintech, China), and Rabbit polyclonal CD163 antibody (Abcam, USA), Mouse monoclonal PD-L1 antibody (Abcam, USA), Rabbit polyclonal β-actin antibody (Abcam, USA) were used for the immunoreactivity. The membranes were stained using an ECL kit, and the expression levels were visualized under enhanced chemiluminescence.

### Enzyme-linked immunosorbent assay (ELISA)

Cell supernatants were collected for ELISA, ELISA assays were performed in 96-well ELISA plates using IL-10, TGF-β2, CCL17 and CCL22 ELISA kit (MultiSciences, China), according to the manufacturers’ instructions.

### Transwell migration assay

Transwell migration assay was performed in 24-well transwell plates with 8-µm pore-size chambers (Corning, USA). Cells were added to the upper chamber at a density of 4 × 10^4^ cells per well, the lower chamber was placed the supernatant of each treatment group and incubated for 24 h. The cells penetrated the membrane were observed under a microscope and counted by Image J.

### Fluorescence in situ hybridization (FISH)

Slides were deparaffinized in xylene and ethanol solutions. Following prehybridization in PBS with 0.5% Triton X-100, the slides were hybridized with probes specific for the junction sites of circWWC3 overnight at 37 °C. The fluorescence signal of circWWC3 was detected by CyTM5-Streptavidin Conjugate (ZyMAXTM Grade, Invitrogen). The nuclei were counterstained by DAPI and the images were taken under a ZEISS LSM 710 confocal microscope (Germany).

### Immunofluorescence

The slides were baked at 60 °C for 30 min, deparaffinized with xylene and dehydrated with ethanol. Antigen retrieval in Tris-EDTA buffer (pH = 9.0) at high pressure for 5 min, followed by cooling down at room temperature naturally. Nonspecific antigen was blocked by 5% BSA, all slides were then incubated with rabbit polyclonal CD163 antibody (Abcam, USA) and mouse monoclonal PD-L1 antibody (Abcam, USA) overnight at 4 °C. After rinsing with PBST, the slices were incubated with fluorescent secondary antibody at 37 °C for 1 h. The slides were counterstained with DAPI at room temperature for 15 min, then analysed using a ZEISS LSM 710 confocal microscope (Germany).

### HE staining and immunohistochemistry (IHC)

For HE staining, tissues were paraffin-embedded, dewaxed, dehydrated, and stained with hematoxylin and eosin. IHC staining were performed as previously described. Mouse IL-4 polyclonal antibody (proteintech, China) was used for IHC analysis. Staining was visualized using a microscope and evaluated by at least two pathologists.

### T cell activation assay

Pan CD3^+^-Tcell extraction kit (Miltenyi Biotec) was used to isolate breast cancer patient-derived T cells. After isolated, using T cells of ImmunoCult Human CD3/CD28/CD2 T cell activator (STEMCELL Technologies) and 100 ng/ml human IL-2 and 50 µM β-mercaptoethanol to expand the CD3 + T cells. T cells were cultured in RPMI with 5% FBS, 500 mM sodium pyruvate, and 500 mM non- essential amino acids (T cell media). HMDMs were isolated from the breast cancer donors. Briefly, monocytes from peripheral blood were cultured in 24-well plate to adhere for 3 days with M-CSF medium. Then, the hMDMs were resuspended and cultured with complete medium for another 2 days. On day 6, hMDMs were stimulated for 48 h with cicWWC3 overexpression medium. On day 7, T cells were reactivated with complete culture medium supplemented with 10 µg/ml of IL-2 (Peprotech) and 20 µg/ml of IL-7 (Peprotech). On Day 8, hMDMs and T cells were co-cultured for 3 days in T cell media. On Day 11, the supernatants were collected to analyze the secretion of IFN-γ and perforin using ELISA.

### Statistical analysis

All statistical analysis were performed with SPSS 22.0 (SPSS Inc, Chicago, USA). The experiments repeated three times. The quantitative data were presented as Mean ± SEM, and analyzed by Student’s t-test. The clinical pathological analysis was performed by Chi-square test. The survival analysis was evaluated by Kaplan-Meier method. *P* value < 0.05 was considered statistically significant.

## Results

### CircWWC3 upregulates the expression of IL-4 in breast cancer cells

**Fig. 1:**
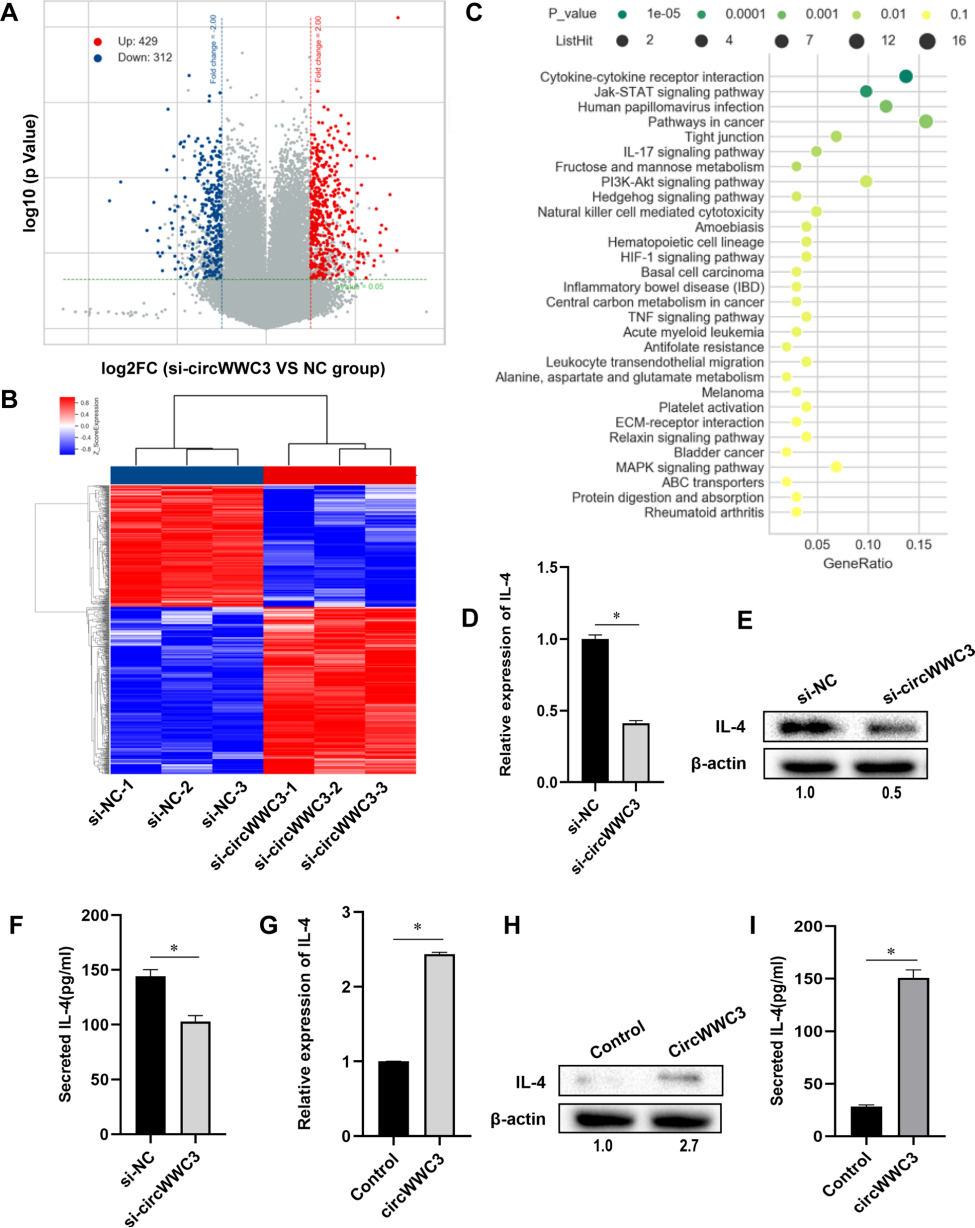
CircWWC3 upregulates the expression of IL-4 in breast cancer cells. **A** Volcano plot showing differentially expressed genes after knocking down circWWC3 in MDA-MB-231 cells by Agilent SurePrint G3 Human Gene Expression v3 8 × 60 K Microarray. The red and blue dots represent the up- and down-regulated genes by FC > 2 and *P* < 0.01. **B** Heat map of hierarchical clustering indicates the up-regulated (red) and down-regulated (blue) genes. **C** Signaling pathways of circWWC3-regulated genes analyzed by KEGG. **D** and **E** qRT-PCR and western blot showed the expression of IL-4 in MDA-MB-231 cells, *P < 0.05. The numbers in western bolt means the ratio of IL-4 to β-actin. **F** ELISA analysis showed the secretion of IL-4 of MDA-MB-231 cells, *P < 0.05. **G** and **H** qRT-PCR and western blot showed the expression of IL-4 in MDA-MB-453 cells, *P < 0.05. **I** ELISA analysis showed the secretion of IL-4 of MDA-MB-453 cells, *P < 0.05

Our previous studies showed that circWWC3 increased the growth and metastasis of breast cancer [14]. For further investigating the molecular mechanism of circWWC3 in breast cancer progression, we performed the microarray analysis after knocking down circWWC3 in breast cancer MDA-MB-231 cells. As shown in Fig. 1A and B and 429 genes are up-regulated and 312 genes are down-regulated after knocking down circWWC3. Most of these genes are involved in thirty pathways, two top of which are cytokine-cytokine receptor interaction and Jak-STAT signaling pathway (Fig. 1C). IL-4 is one of most relevant candidates that are involved in these pathways, and is down-regulated after si-circWWC3 transfection. QRT-PCR and western blot results confirmed that siRNA-mediated knockdown of circWWC3 inhibited the expression of IL-4 at both mRNA and protein levels (Fig. 1D and E). ELISA result suggested that the secreted IL-4 was also inhibited after suppressing circWWC3 (Fig. 1F). In contrast, overexpression of circWWC3 increased the expression and secretion of IL-4 in breast cancer MDA-MB-453 cells which has low expression of circWWC3 (Fig. 1G–I). Taken together, our results suggested that circWWC3 upregulates the expression and secretion of IL-4 in breast cancer cells.

### CircWWC3 facilitates M2-like TAM polarization through up-regulating IL-4 expression and secretion to promote the migration of breast cancer cells

**Fig. 2:**
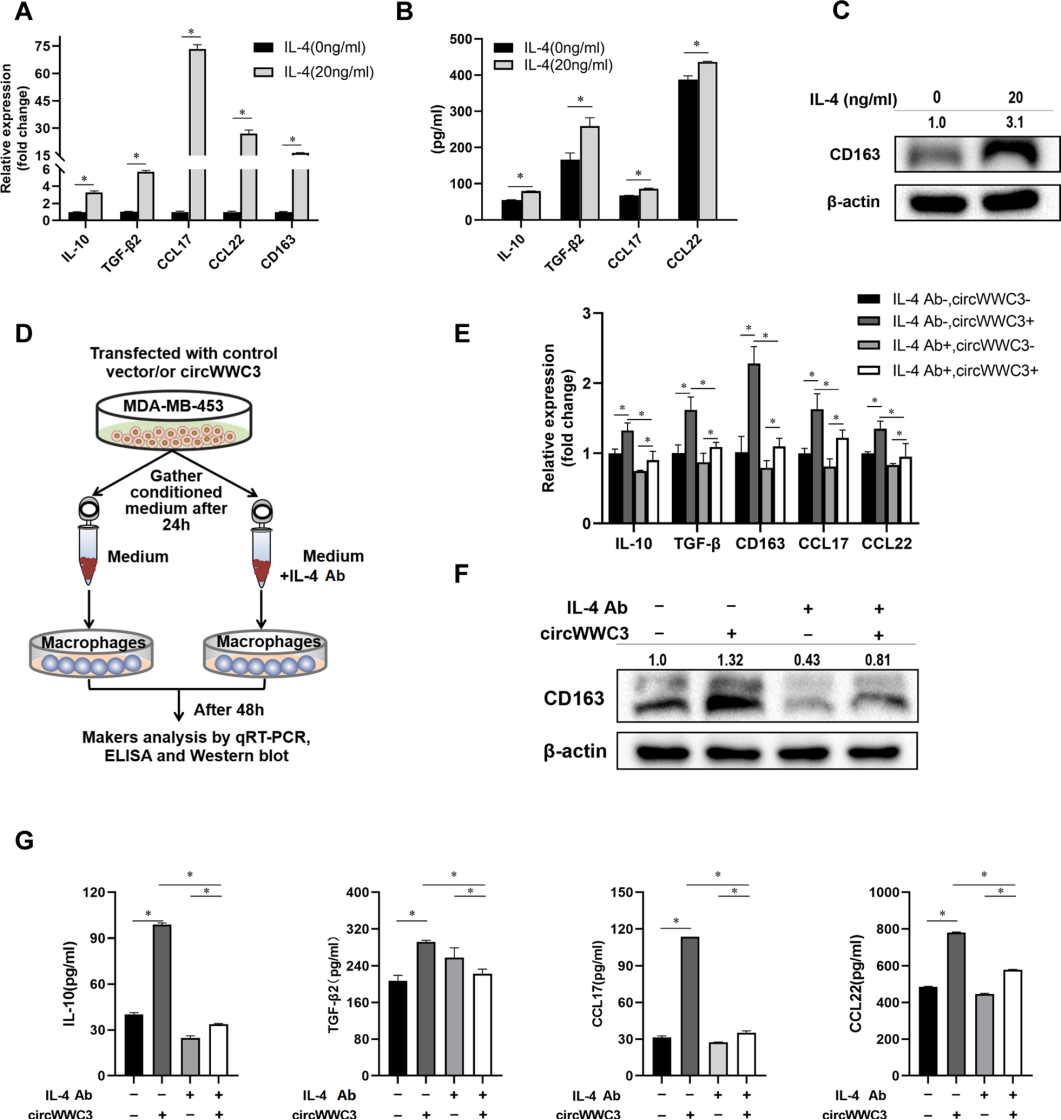
Over-expression of circWWC3 promotes M2-like TAM polarization through up-regulating IL-4 expression and secretion. **A**–**C** qRT-PCR, western blot, and ELISA showed the expression of M2 markers and chemokines in THP-1 cells, *P < 0.05. The numbers in western bolt means the ratio of CD163 to β-actin. **D**–**G** qRT-qPCR, western blot, and ELISA showed the expression of M2 macrophage-associated markers and chemokines in THP-1 cells after being co-cultured with the supernatant of transfected MDA-MB-453, *P < 0.05

**Fig. 3:**
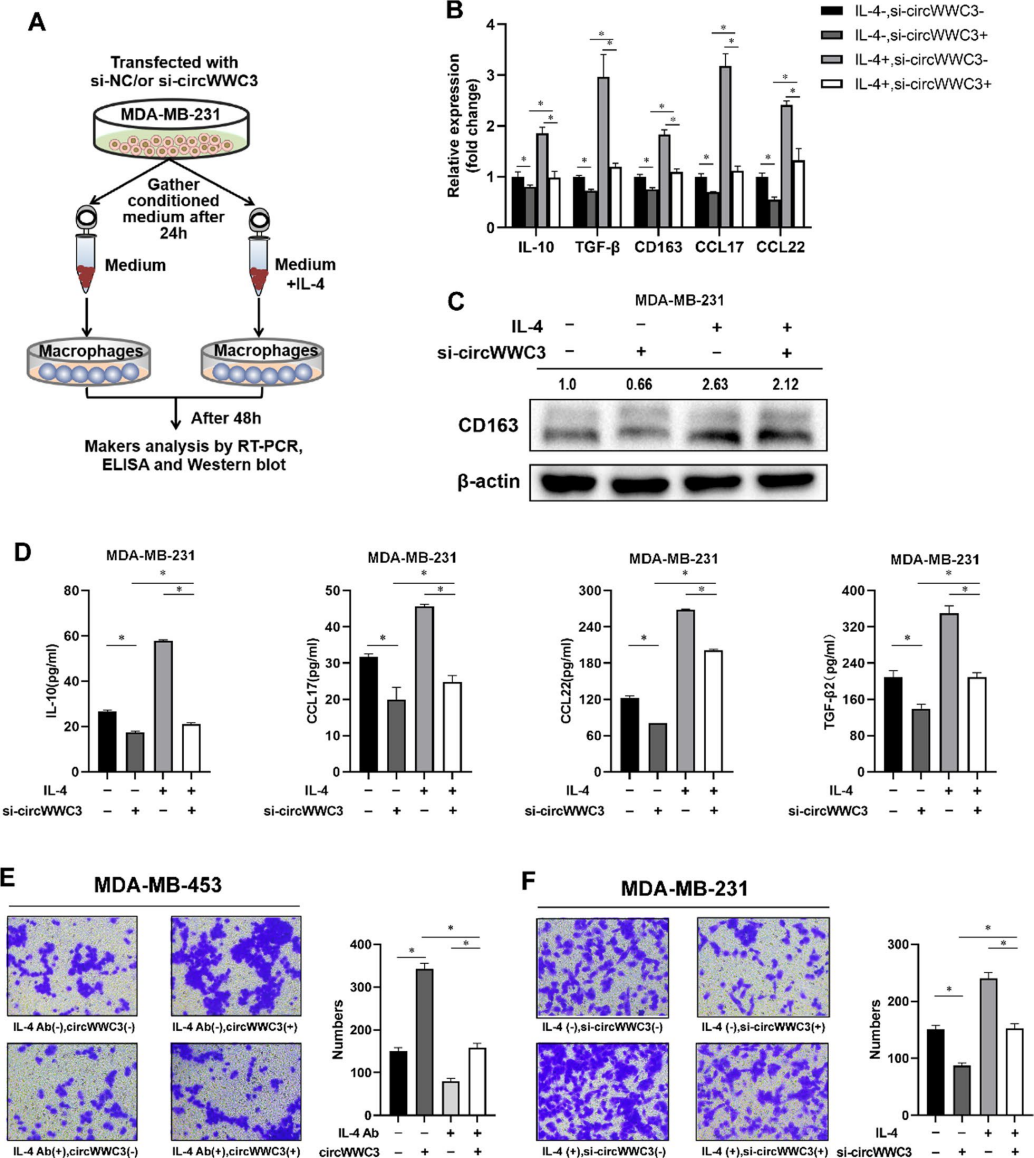
siRNA-mediated knockdown of circWWC3 inhibits M2-like TAM polarization through down-regulating IL-4 expression and secretion. **A**–**D** qRT-qPCR, western blot, and ELISA showed the expression of M2 macrophage-associated markers and chemokines in THP-1 cells after being co-cultured with the supernatant of transfected MDA-MB-231, *P < 0.05. **E** Cell migration assay showed the migration MDA-MB-453 cells after being co-cultured with the supernatant of conditioned THP-1 cells, *P < 0.05. **F** Cell migration assay showed the migration MDA-MB-231 cells after being co-cultured with the supernatant of conditioned THP-1 cells, *P < 0.05

IL-4 was considered to induce M2-like phenotypes of mononuclear cells. In our study, IL-4 could induce the expression of M2 markers including IL-10, TGF-β, and CD163 as well as some chemokines including CCL-17 and CCL-22 in human myeloid leukemia mononuclear THP-1 cells (Fig. 2A–C). To elucidate whether circWWC3-mediated IL-4 secretion affects macrophage alternative polarization in breast cancer microenvironment, THP-1 cells were co-cultured with breast cancer cells-conditioned medium, then qRT-PCR, western blot and ELISA were performed to detect the changes of M2 markers as well as chemokines (Figs. 2D and 3A). After being cultured with breast cancer MDA-MB-453 cell-conditioned medium, macrophages were inclined to the M2 phenotype (IL-10 high, TGF-β high, CD163 high, CCL-17 high, CCL-22 high) in the circWWC3-transfected group as compared to the control group (Fig. 2E and G). After adding IL-4 neutralizing antibody (IL-4 Ab) in medium of the circWWC3-transfected group, this effect has disappeared (Fig. 2E and G). In the si-circWWC3-transfected MDA-MB-231 cells, the M2 markers and chemokines seemed to be decreased (Fig. 3A–D). After adding IL-4 cytokine in medium of the si-circWWC3-transfected cells, this effect has disappeared (Fig. 3A–D).

To further explore the effect of circWWC3-mediated M2-like TAM polarization on the function of breast cancer cells, we detected the migration ability of breast cancer cells under different conditions. As shown in Fig. 3E, the migration ability of MDA-MB-453 cells was increased upon stimulation with conditioned supernatants from circWWC3-overexpressed breast cancer medium-stimulated THP-1 cells, whereas IL-4 neutralizing antibody could block the increase of conditioned supernatants-induced migration of MDA-MB-453 cells. The migration of MDA-MB-231 cells was decreased upon stimulation with conditioned supernatants from si-circWWC3-transfected breast cancer medium-stimulated THP-1 cells, whereas IL-4 cytokine could neutralize the decrease of conditioned supernatants-induced migration of MDA-MB-231 cells (Fig. 3F). Taken together, our results indicated that circWWC3 could induce M2-like TAM polarization through up-regulating IL-4 expression and secretion to promote the migration of breast cancer cells.

### CircWWC3 enhances PD-L1 expression of TAMs and breast cancer cells through up-regulating IL-4 expression and secretion to facilitate breast cancer immune escape

**Fig. 4:**
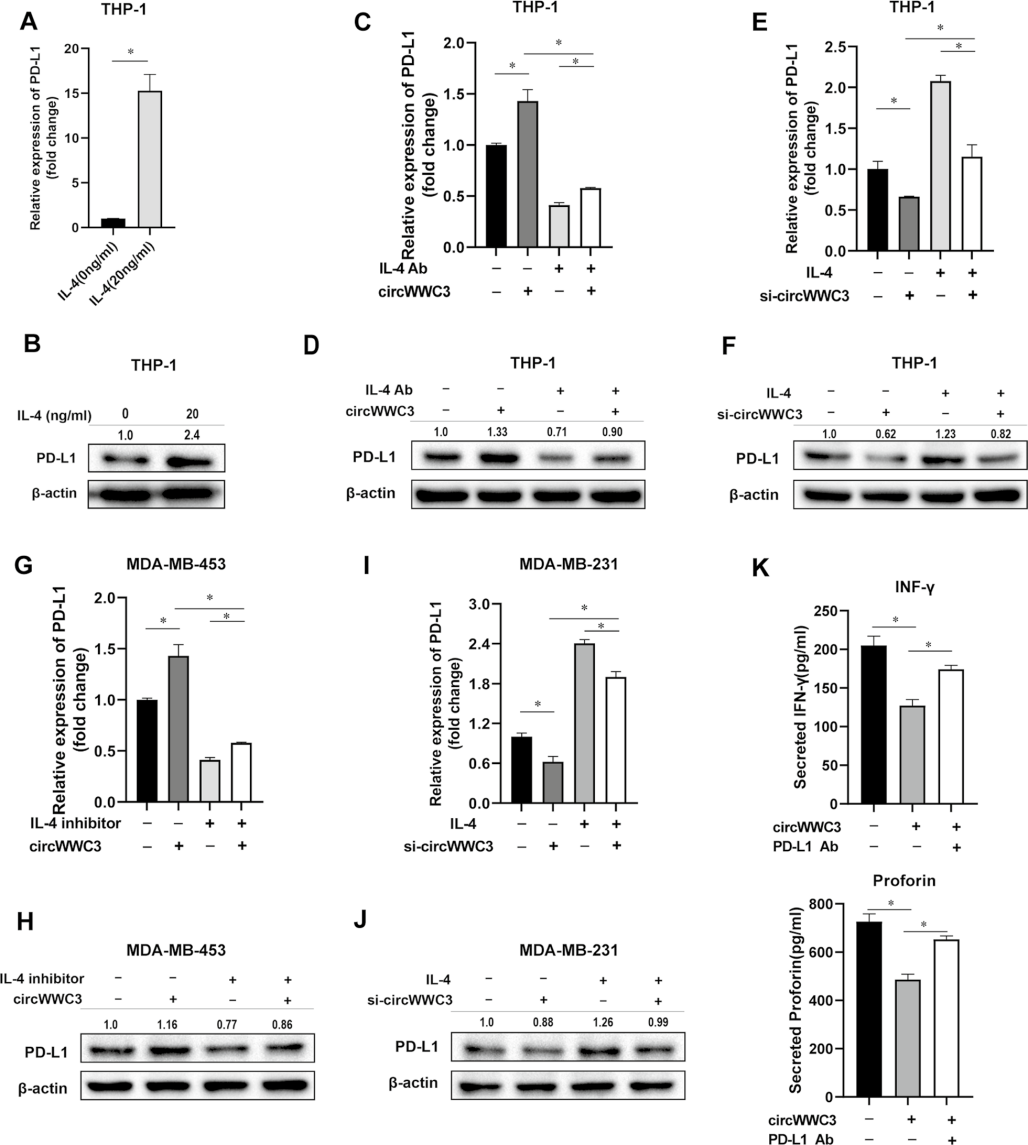
CircWWC3 enhances PD-L1 expression of TAMs and breast cancer cells through up-regulating IL-4 expression and secretion to facilitate breast cancer immune escape. **A**, **B** qRT-PCR and western blot showed the expression of PD-L1 in THP-1 cells after treated with IL-4, *P < 0.05. The numbers in western bolt means the ratio of PD-L1 to β-actin. **C**–**F** qRT-PCR and western blot showed the expression of PD-L1 in THP-1 cells after being co-cultured with the supernatant of transfected MDA-MB-231, *P < 0.05. **G**–**J** qRT-PCR and western blot showed the expression of PD-L1 in MDA-MB-453 and MDA-MB-231 cells under different conditions, *P < 0.05.** K** ELISA assay showed the secretion of INF-γ and Proforin from activated T cells at the presence of circWWC3-conditioned medium and PD-L1 antibody, *P < 0.05

IL-4 was reported to increase the expression of PD-L1 of TAMs. In our present study, we also observed the induction of PD-L1 at both mRNA and protein levels in TMAs after IL-4 treatment (Fig. 4A, B). In the rescue experiments, circWWC3-transfected MDA-MB-453 medium induced the expression of PD-L1 in TAMs, while adding IL-4 neutralizing antibody in medium inhibited circWWC3-mediated induction of PD-L1 at both mRNA and protein levels (Fig. 4C, D). In the si-circWWC3-transfected group, the expression of PD-L1 in TAMs was suppressed, while adding IL-4 cytokine in medium reversed circWWC3-mediated suppression of PD-L1 at both mRNA and protein levels (Fig. 4E, F).

We also detected the expression of PD-L1 in breast cancer cells. After transfected circWWC3, the expression of PD-L1 in MDA-MB-453 cells were increased, whereas IL-4 inhibitor (IL-4 inhibitor (Suplatast Tosilate, IPD 1151 T, a cytokine inhibitor which can inhibit IL-4 expression by cells) inhibited circWWC3-mediated induction of PD-L1 at both mRNA and protein levels (Fig. 4G, H). In MDA-MB-231 cells, siRNA-mediated knock down of circWWC3 inhibited the expression of PD-L1, while IL-4 cytokine recovered si-circWWC3-mediated suppression of PD-L1 (Fig. 4I, J). Next, we validated the functionality of circWWC3-mediated PD-L1 up-regulation by performing a T cell activation assay with breast cancer patient-derived T cells co-cultured for 3d with autologous monocyte-derived macrophages polarized with circWWC3-conditioned medium. As shown in Fig. 4K, the secretion of perforin and IFN-γ from activated T cells were significantly suppressed at the presence of circWWC3-conditioned medium, which can be recovered by PD-L1 antibody, suggesting that circWWC3-mediated up-regulation of PD-L1 in TAMs suppressed T cells activation. Taken together, our results indicated that circWWC3 could enhance PD-L1 expression of TAMs and breast cancer cells through up-regulating IL-4 expression and secretion to facilitate breast cancer immune escape.

### Correlation between circWWC3, IL-4, as well as PD-L1 expression and M2-like TAM polarization in breast cancer microenvironment

**Fig. 5:**
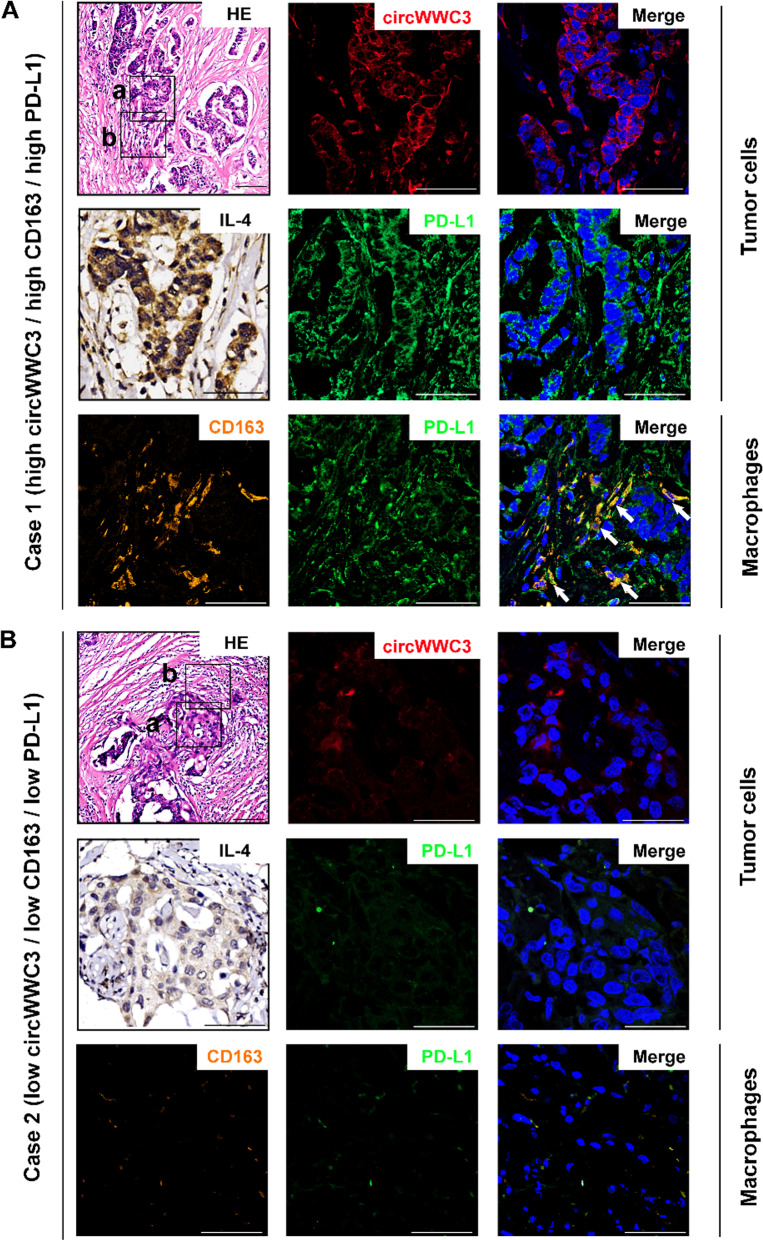
The expression of circWWC3, IL-4, PD-L1, and CD163 in breast cancer tissues. circWWC3 (red) was detected by FISH. CD163 (orange) and PD-L1 (green) were detected by Immunofluorescence. IL-4 (brown) was detected by IHC

To further detect the expression status of circWWC3 in breast cancer tissues, and explore its correlation with M2-like TAM polarization and PD-L1 expression, we firstly performed FISH staining to detect the expression of circWWC3 in 140 cases of breast cancer tissues by using the probe targeting the junction site of circWWC3. Then, we detected the CD163 and PD-L1 expression by using immunofluorescence staining, and detected IL-4 expression by using IHC staining. CD163 positive cells were considered as M2-type macrophages. As shown in Fig. 5A, in circWWC3 high-expressed breast cancer tissues, the expression of IL-4 and PD-L1 in breast cancer cells was also high, which suggested the possible positive correlation between circWWC3 and IL-4 as well as PD-L1 in breast cancer cells in vivo. In circWWC3 high-expressed breast cancer tissues, M2-type macrophages in breast cancer microenvironment were relatively increased with high expression of PD-L1, indicating that high expression of circWWC3 facilitates M2-like TAM polarization and PD-L1 expression of TAMs (Fig. 5A). By contrast, in circWWC3 low-expressed breast cancer tissues, the expression of IL-4 and PD-L1 was low, and M2-type macrophages in breast cancer microenvironment were relatively decreased (Fig. 5B). Statistically analysis revealed that circWWC3 expression is positively correlated with IL-4 and PD-L1 expression as well as M2-like TAM polarization (Additional file 1: Table S2).

**Fig. 6:**
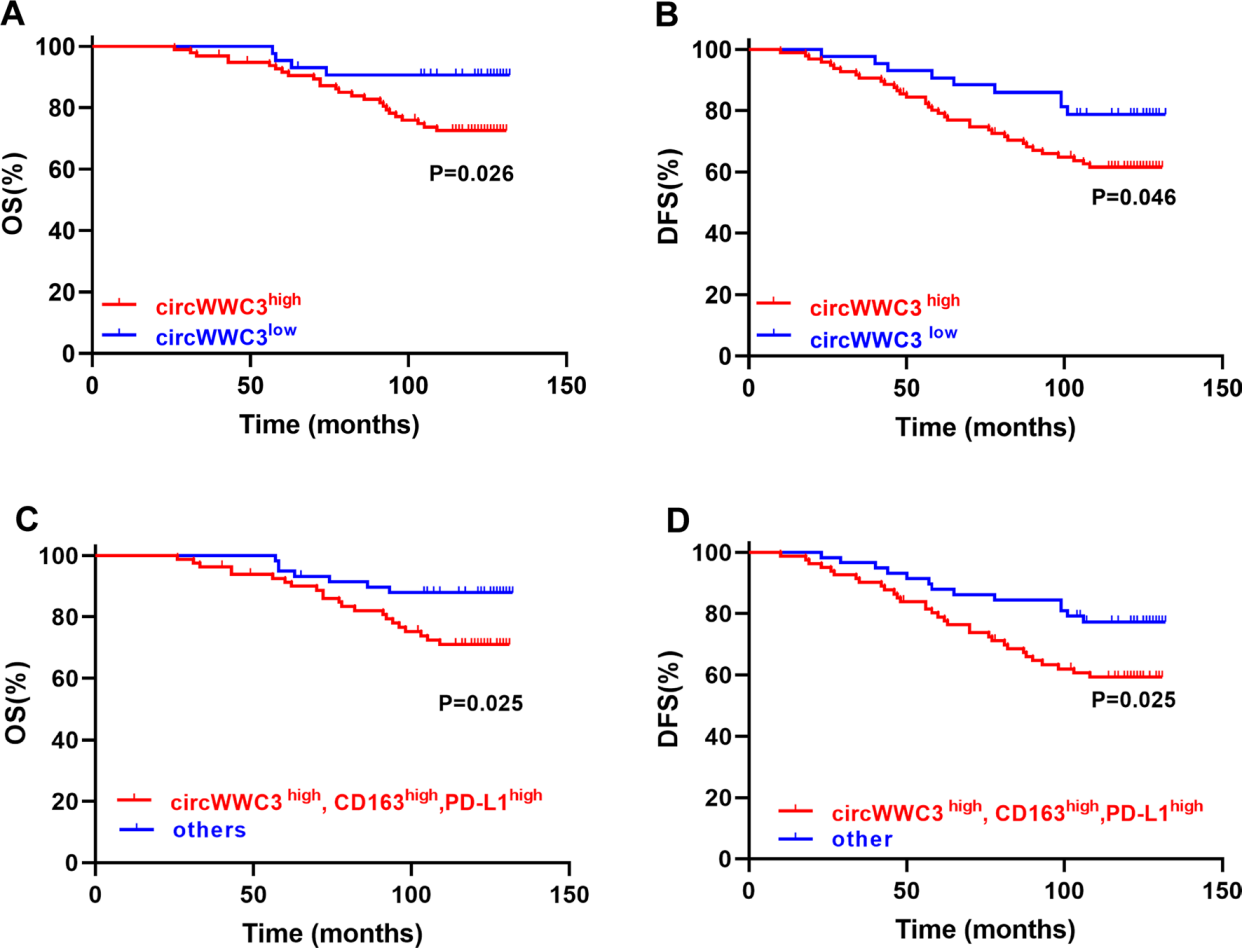
**A**, **B** High expression of circWWC3 was associated with the poor overall survival and disease-free survival of breast cancer patients. **C**, **D** Breast cancer patients with circWWC3^high^/PD-L1^high^ breast cancer cells and CD163^high^ macrophages had a poorer overall survival and disease-free survival

**Fig. 7:**
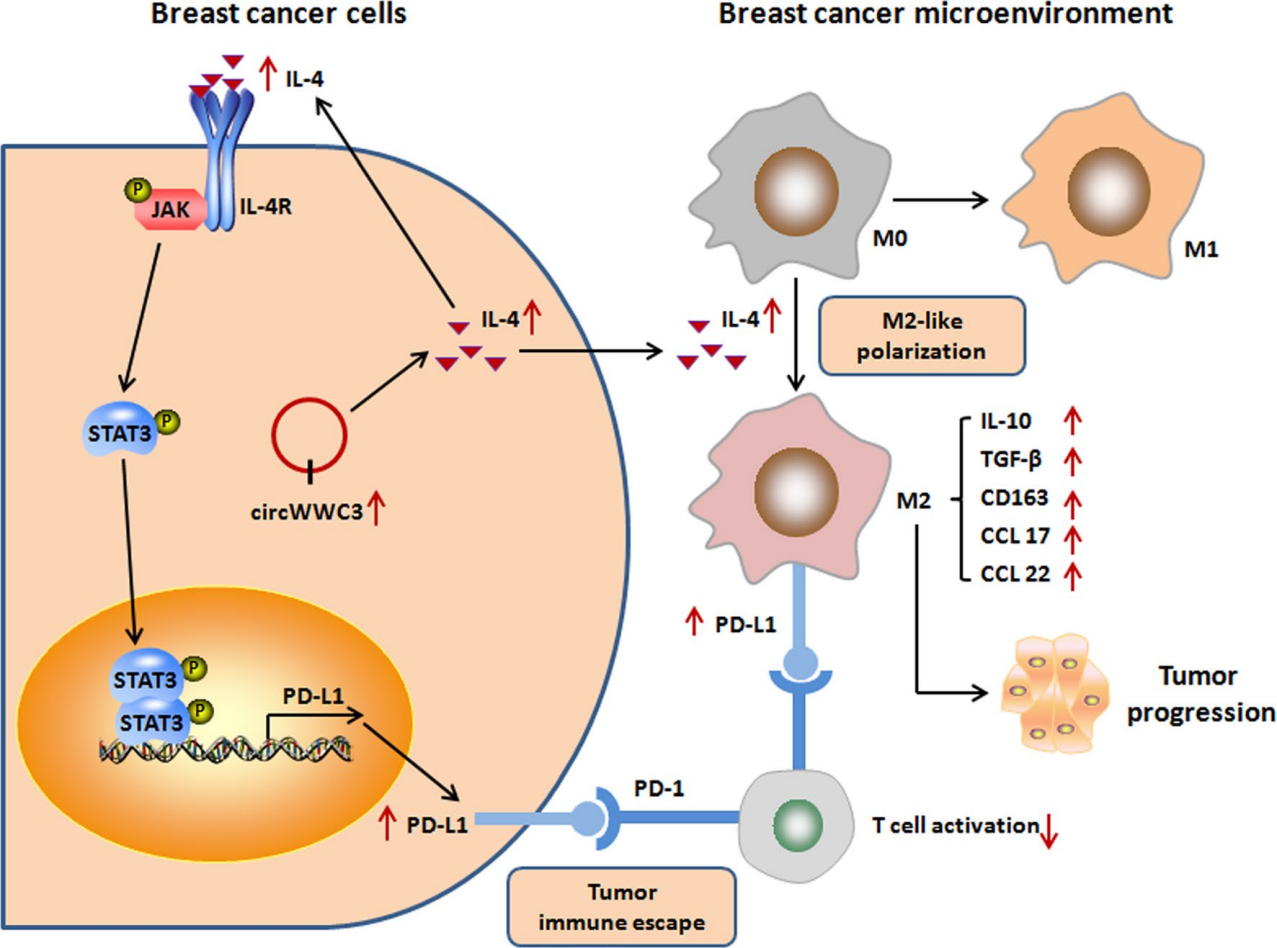
The molecular mechanism diagram that circWWC3 augments breast cancer progression through promoting M2 macrophage polarization and tumor immune escape via regulating the expression and secretion of IL-4

For further explore the clinical significance of circWWC3 expression in breast cancer, we analyzed its correlation with the overall survival and disease-free survival of breast cancer patients. As shown in Fig. 6A and B, high expression of circWWC3 was associated with shorter overall survival and disease-free survival of in 140 cases of breast cancer patients. Breast cancer patients with circWWC3^high^/PD-L1^high^ breast cancer cells and CD163^high^ macrophages had a poorer overall survival and disease-free survival (Fig. 6C, D). Taken together, our results indicated that circWWC3 promotes breast cancer progression through facilitating M2-like TAM polarization and PD-L1 expression of TAMs as well as breast cancer cells via regulating IL-4 expression in vivo (Fig. 7).

## Discussion

Our previous study demonstrated that circular RNA circWWC3 promoted breast cancer progression and metastasis [14]. To further understand mechanistically how circWWC3 facilitates breast cancer metastasis, we knocked down the expression of circWWC3 in MDA-MB-231 cells and performed microarray analysis. Our results showed that IL-4 was significantly reduced after suppressing circWWC3, suggesting that circWWC3 directly or indirectly regulated IL-4 expression.

TAMs have been widely recognized as a favorable condition for tumor progression, including tumor cell growth, Epithelial-Mesenchymal Transition (EMT), and immune suppression in TME. In TME, TAMs were mixed with the M1 and M2 phenotypes, which played opposite roles in tumor progression. M2-like TAMs markers included IL-10, TGF-β, CD163, and some chemokines such as CCL-17 and CCL-22. IL-4 is mainly supplied by CD4^+^ T cells. Tumor cells also secrete IL-4 [15, 16]. As a major activator of TAM phenotypes in tumor microenvironment, IL-4 supplied by either T cells or tumor cells can act on TAMs to up-regulate cathepsin enzyme activity in TAMs and augment the EGF/CSF-1 paracrine loop between TAMs and tumor cells [17, 18]. Furthermore, TAMs release numerous cytokines and chemokines with distinct pro-tumorigenic properties [19]. These effects of IL-4 collectively prime TAMs with the capability to promote tumor growth and progression [20–22]. The expression and secretion of IL-4 in tumor cells were regulated by various factors. MiR-195-5p was reported to inhibit NOTCH2 expression and activation in a post-transcriptional manner, leading to the down-regulation of NOTCH2/GATA3-mediated IL-4 secretion in colorectal cancer cells, ultimately suppressing M2-like TAM polarization and tumor progression [15]. By using qRT-PCR, western blot and ELISA assay, we confirmed that circWWC3 could up-regulate the expression and secretion of IL-4 in breast cancer cells. Furthermore, circWWC3 was positively correlated with the expression of IL-4 in breast cancer tissues. Taken together, our results indicated that circWWC3 is an activator of IL-4. However, the molecular mechanism how circWWC3 regulates IL-4 expression and secretion is still unknown.

Since circWWC3 up-regulates the expression and secretion of IL-4 in breast cancer cells, we postulated that circWWC3-mediated up-regulation of IL-4 secretion might affect the acquisition of tumor-promoting phenotypes of TAMs in breast cancer microenvironment. Through co-cultured with breast cancer cells-conditioned medium, THP-1 cells were confirmed to incline to M2 phenotype at the present of circWWC3 at an IL-4-dependent manner. Moreover, circWWC3-conditioned M2-like TAM polarization promoted migration of breast cancer cells. These results indicated that circWWC3 induces M2-like TAM polarization through up-regulating IL-4 expression and secretion to promote the migration of breast cancer cells.

As we know, TAMs express PD-L1 and contribute to the immune-suppressive tumor microenvironment. In a screen of several different human tumors, PD-L1-expressing macrophages were more abundant than PD-L1-expressing tumor cells [23]. Even in models where PD-L1 was not expressed in the tumor cells themselves, PD-L1 antibody treatment could induce antitumor activity, indicating that PD-L1 expression by macrophages may be a key element driving response to PD-L1 antibody treatment[24, 25]. It has been reported that blocking PD-L1 signals can trigger macrophage proliferation, survival, activation, and antitumor activity in tumor tissues [26]. IL-4 could increase the expression of PD-L1 through JAK-STAT pathway in both TAM and tumor cells [27–30]. In our study, circWWC3 could enhance PD-L1 expression in TAMs and breast cancer cells through up-regulating IL-4 expression and secretion. At in vivo condition, high expression of circWWC3 was associated with more TAMs in breast cancer environment, and was correlated with high PD-L1 expression in TAMs and tumor cells. Furthermore, breast cancer patients with circWWC3^high^/PD-L1^high^ breast cancer cells and CD163^high^ macrophages had a poorer overall survival and disease-free survival. However, in our present study, we did not find any correlation between circWWC3 expression and the molecular subtype of breast cancer (Data not shown).

In summary, our study suggested that IL-4 could promote M2 macrophage polarization and PD-L1 expression of TAMs and breast cancer cells. CircWWC3 could augment breast cancer progression through promoting M2 macrophage polarization and tumor immune escape via regulating the expression and secretion of IL-4. Our study indicated the potential importance of targeting circWWC3 to suppress breast cancer progression.

## Supplementary Information

**Additional file 1: Table S1.**. The primers sequences. **Table S2.** Association between the expression of circWWC3 and the expression of IL-4, CD163 as well as PD-L1 (breast cancer cells). **Figure S1.** The basic expression of circWWC3 and IL-4 in breast cancer cells. A, qRT-PCR showed the expression of circWWC3 in MDA-MB-453 and MDA-MB-231 cells. B, qRT-PCR showed the expression of IL-4 in MDA-MB-453 and MDA-MB-231 cells. C, ELISA analysis showed the secretion of IL-4 of MDA-MB-453 and MDA-MB-231 cells.

## Data Availability

All data generated that are relevant to the results presented in this article are included in this article. Other data that were not relevant for the results presented here are available from the corresponding author Dr. Sang upon reasonable request.
