# Supplementary material for: Circular RNA circWWC3 augments breast cancer progression through promoting M2 macrophage polarization and tumor immune escape via regulating the expression and secretion of IL-4
Source: Cancer Cell Int. 2022 Aug 22;22:264. doi: 10.1186/s12935-022-02686-9 (PMC9396792; doi:10.1186/s12935-022-02686-9)
Supplement: Supplementary file 1 — Additional file 1: Table S1.. The primers sequences. Table S2. Association between the expression of circWWC3 and the expression of IL-4, CD163 as well as PD-L1 (breast cancer cells). Figure S1. The basic expression of circWWC3 and IL-4 in breast cancer cells. A, qRT-PCR showed the expression of circWWC3 in MDA-MB-453 and MDA-MB-231 cells. B, qRT-PCR showed the expression of IL-4 in MDA-MB-453 and MDA-MB-231 cells. C, ELISA analysis showed the secretion of IL-4 of MDA-MB-453 and MDA-MB-231 cells. [file 12935_2022_2686_MOESM1_ESM.docx]

**Additional Tables**

**Table S1 The primers sequences**

| **Genes** | **Primers** |
| --- | --- |
| ***IL-4*** | forward, 5'-AACAGCCTCACAGAGCAGAA-3’  reverse, 5'-GTGTTCTTGGAGGCAGCAAA-3' |
| ***IL-10*** | forward, 5'-GTTCTTTGGGGAGCCAACAG-3’  reverse, 5'-GCTCCCTGGTTTCTCTTCCT-3' |
| ***TGF-β2*** | forward, 5’-GAGTCACAACAGACCAACCG-3’  reverse, 5'-GGCAGCAATTATCCTGCACA-3’ |
| ***CCL17*** | forward, 5’-ACTGTCTCCCGGGACTACCT-3’  reverse, 5'-TTTAATCTGGGCCCTTTGTG-3’ |
| ***CCL-22*** | forward, 5’-TGTGCCAACTCTCTGCATTC-3’  reverse, 5'-GACCTCAGCTTAGGGCAGTG-3’ |
| ***PD-L1*** | forward, 5'- CGTTGTGCTTGAACCCTTGA-3’  reverse, 5'- ACACAAGGAGCTCTGTTGGA-3' |
| ***CD163*** | forward, 5'-ACGCCAGTAAGGGATTTGGA-3’ |
|  | reverse, 5'-GTCACGCCAGCATCTTCATT-3' |
| ***GAPDH*** | forward, 5’-AGCCACATCGCTCAGACAC-3’  reverse, 5’-GCCCAATACGACCAAATCC-3’ |

**Table S2 Association between the expression of circWWC3 and the expression of IL-4, CD163 as well as PD-L1 (breast cancer cells)**

|  | | **IL-4** | | **P** | **CD163** | | **P** | **PD-L1** | | **P** |
| --- | --- | --- | --- | --- | --- | --- | --- | --- | --- | --- |
|  |  | **Low** | **High** |  | **Low** | **High** |  | **Low** | **High** |  |
| **circWWC3** | **Low** | 34 | 9 | 0.000 | 37 | 6 | 0.000 | 33 | 10 | 0.000 |
|  | **High** | 6 | 91 |  | 9 | 88 |  | 40 | 90 |  |

**Additional Figure**


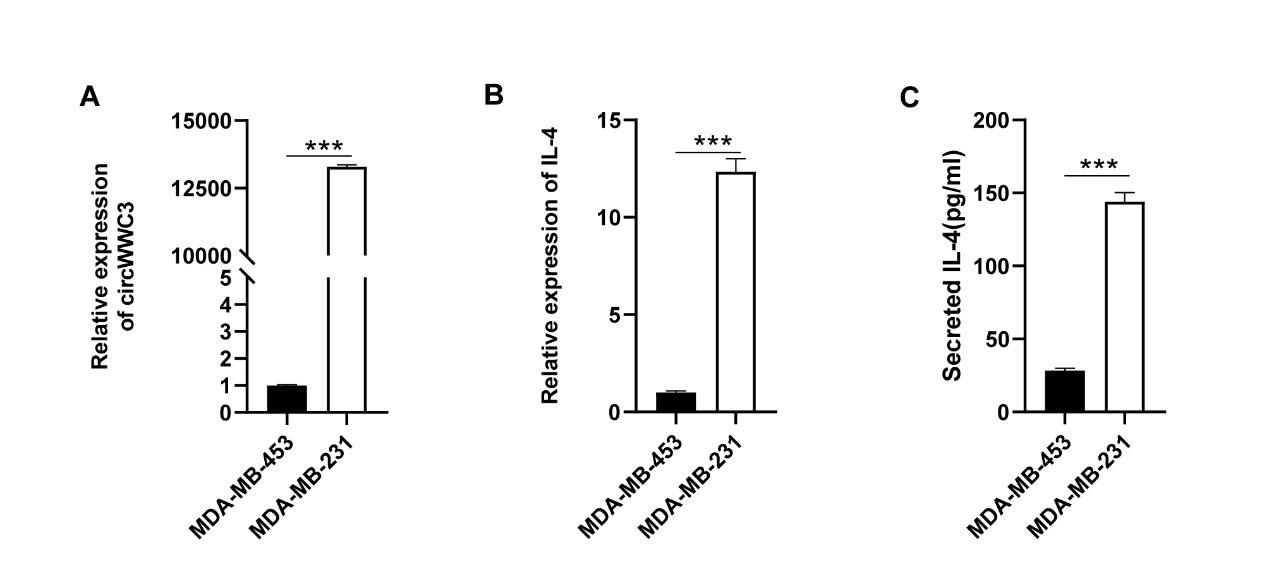

**FIGURE S1 The basic expression of circWWC3 and IL-4 in breast cancer cells.**

A, qRT-PCR showed the expression of circWWC3 in MDA-MB-453 and MDA-MB-231 cells. B, qRT-PCR showed the expression of IL-4 in MDA-MB-453 and MDA-MB-231 cells. C, ELISA analysis showed the secretion of IL-4 of MDA-MB-453 and MDA-MB-231 cells.
